# Construction of a circadian rhythm-related gene signature for predicting the prognosis and immune infiltration of breast cancer

**DOI:** 10.3389/fmolb.2025.1540672

**Published:** 2025-02-06

**Authors:** Lin Ni, He Li, Yanqi Cui, Wanqiu Xiong, Shuming Chen, Hancong Huang, Zhiwei Wang, Hu Zhao, Bing Wang

**Affiliations:** ^1^ Department of General Surgery, Fuzong Clinical Medical College of Fujian Medical University, 900TH Hospital of Joint Logistics Support Force, PLA, Fuzhou, China; ^2^ Department of General Surgery, Fuzhou General Teaching Hospital, Fujian University of Traditional Chinese Medicine, 900TH Hospital of Joint Logistics Support Force, Fuzhou, China; ^3^ Department of Cardiothoracic surgery, Fuzong Clinical Medical College of Fujian Medical University, 900TH Hospital of Joint Logistics Support Force, PLA, Fuzhou, China; ^4^ Department of General Surgery, Dongfang Hospital of Xiamen University, School of Medicine, Xiamen University, 900TH Hospital of Joint Logistics Support Force, Fuzhou, China

**Keywords:** breast cancer, circadian rhythm, machine learning, a risk model, predict prognosis

## Abstract

**Objectives:**

In this study, we constructed a model based on circadian rhythm associated genes (CRRGs) to predict prognosis and immune infiltration in patients with breast cancer (BC).

**Materials and methods:**

By using TCGA and CGDB databases, we conducted a comprehensive analysis of circadian rhythm gene expression and clinicopathological data. Three different machine learning algorithms were used to screen out the characteristic circadian genes associated with BC prognosis. On this basis, a circadian gene prediction model about BC prognosis was constructed and validated. We also evaluated the association of the model’s risk score with immune cells and immune checkpoint genes, and analyzed prognostic genes and drug sensitivity in this model.

**Results:**

We screened 62 DEGs, including 30 upregulated genes and 32 downregulated genes, and performed GO and KEGG analysis on them. The above 62 DEGs were included in Cox analysis, LASSO regression, Random Forest and SVMV-RFE, respectively, and then the intersection was used to obtain 5 prognostic related characteristic genes (SUV39H2, OPN4, RORB, FBXL6 and SIAH2). The Risk Score of each sample was calculated according to the expression level and risk coefficient of 5 genes, Risk Score= (SUV39H2 expression level ×0.0436) + (OPN4 expression level ×1.4270) + (RORB expression level ×0.1917) + (FBXL6 expression level ×0.3190) + (SIAH2 expression level × -0.1984).

**Conclusion:**

SUV39H2, OPN4, RORB and FBXL6 were positively correlated with Risk Score, while SIAH2 was negatively correlated with Risk Score. The above five circadian rhythm genes can construct a risk model for predicting the prognosis and immune invasion of BC.

## 1 Introduction

Breast cancer is the tumor with the highest incidence among females and has seriously threatened the health of modern women (Siegel et al., 2023). Breast cancer accounts for over 24% of new cancer cases among global females and approximately 15% of cancer-related deaths (Sun et al., 2022). The 5-year survival rate of primary breast cancer has reached 99%. However, for patients with a non-metastasis interval of <5 years, 5–10 years, and >10 years, the 5-year survival rates of recurrent metastatic breast cancer are 23%, 26%, and 35% respectively (Chang et al., 2019). Nevertheless, the existing treatment modalities still have certain limitations. How to enhance the therapeutic efficacy of patients, assess the prognosis of patients, and explore better treatment strategies is an urgent and challenging task for contemporary medical practitioners.

The 24-h oscillations of an organism are known as circadian rhythms which are set by the biological clock and/or circadian oscillator (Mukherji et al., 2019). Their molecular activity is determined by the circadian clock gene’s rhythmically regulated transcription (Ortega-Campos et al., 2023). The biological clock, driven by a series of transcription and translation cycles, post-translational modification and degradation mechanisms of biological clock genes, produces the circadian rhythm and controls the rhythm of human’s daily biochemical, physiological and behavioral functions, and makes the body’s physiological, biochemical, behavioral and other life activities, which show amplitude oscillations of about 24 h (Malik et al., 2022; Lou et al., 2021). Core clock genes mainly include: Clock (Circadian locomotor output cycles kaput), Bmal1 (Brain and muscle ARNT-like 1), Per1, Per2, Per3, Cry1, Cry2, NPAS2, CKIε, Tim, Rev-Erb and DEC et al. Circadian clock gene Bmal1 can regulate intestinal stem cell pathway, and the loss of Bmal1 or circadian photoperiod may increase the occurrence of tumors (Stokes et al., 2021). BMAL1 and clock have anti-apoptotic effects in promoting the proliferation of liver cancer cells (Qu et al., 2023). Lask et al. implanted human breast cancer xenograft (MCF7) into a nude mouse model and found that night lighting could affect the growth of human breast cancer (Stevens, 2005).

Existing treatments of BC still have certain limitations. It is still an urgent and arduous task to improve the therapeutic effect of BC patients, further understand the prognosis of patients, and explore better treatment strategies. With the development of modern medicine, combined with the assistance of artificial intelligence, bioinformatics combined with machine learning algorithms has been widely used in the research of various diseases. More and more models for screening, diagnosis and prognosis of diseases are being developed. In this study, we constructed a model based on circadian rhythm associated genes (CRRGs) to predict prognosis and immune infiltration in patients with BC.

## 2 Materials and methods

### 2.1 Bioinformatics analysis

The circadian rhythm gene set (comprising 210 genes) was retrieved from the Circadian Genes Database (CGDB) (http://cgdb.biocuckoo.org) on 27 June 2023. Concurrently, the BC RNA-Seq data (TPM values) and clinicopathological data (1,086 cancer samples and 99 adjacent normal tissue samples) were acquired from the TCGA database (https://portal.gdc.cancer.gov). The expression levels of the circadian rhythm genes in BC patients were screened and subjected to log2 transformation. The external validation dataset GSE42568 (104 cancer samples and 17 adjacent normal tissue samples) and GSE58812 (107 cancer samples)was obtained from the GEO (Gene Expression Omnibus) (https://www.ncbi.nlm.nih.gov/geo) database. All the datasets in this study originated from public databases and thus did not require ethical approval.

### 2.2 Screening, mutation and copy number variation of circadian differential genes

The gene mutation data and copy number variation data of BRCA were downloaded from the TCGA database and corresponded to the circadian rhythm genes. They were processed and visualized respectively by the “maftools” and “Rcircos” packages of R-4.3.0 software. The “DESeq2″ package was employed to screen the differentially expressed genes (DEGs) of circadian rhythm with the criteria of P < 0.05 and | log2(FoldChange)| > 1, and the “pheatmap”, “ggplot2″ and “psych” packages were utilized to draw the volcano plot and the heatmap of the correlation between genes and clinical traits.

### 2.3 Functional enrichment analysis and construction of protein-protein interaction networks

The “enrichplot”, “org.Hs.e.g.,.db” and “clusterProfiler” packages were employed to conduct Gene Ontology (GO) functional enrichment analysis, Kyoto Encyclopedia of Genes and Genomes pathway (KEGG) analysis and Gene Set Enrichment Analysis (GSEA) on the DEGs. A P value <0.05 was regarded as statistically significant, and the results were visualized using the “ggplot2″ package. The protein-protein interaction network (PPI) of the differential genes was generated via The STRING website (www.string-db.org) with a minimum correlation score of 0.400 as the threshold. The data was downloaded from the database in TSV format and visualized using Cytoscape_v3.9.0 software.

### 2.4 Construction of risk score prediction model

The expression levels of circadian rhythm DEGs and survival information of 1069 BC samples in the training group were consolidated. Patients without overall survival (OS) and survival status (Censor) were excluded. The remaining 1049 BC samples were successively incorporated into Cox analysis and three machine learning algorithms (LASSO regression, Random Forest, SVM-RFE). For the assessment of the prognostic value in this study, the intersection of DEGs calculated by the four algorithms was selected, and ultimately a risk score (Risk Score) prediction model was constructed. The formula for the risk score prediction model is: Risk Score = Exp1 × C1 + Exp2 × C2 + … + Expn × Cn (Exp: the expression level of the gene, C: the regression coefficient obtained by LASSO regression analysis, n: the number of intersection genes). The Risk Score of each sample was calculated based on the above formula, and the BC samples were classified into low-risk and high-risk groups using the median Risk Score as the cut-off value. A P value <0.05 was considered statistically significant.

### 2.5 Evaluation of the risk prediction model and construction of the nomogram

Firstly, packages such as “survival” and “survminer” were exploited to draw the Kaplan-Meier (K-M) survival curve for comparing the overall survival (OS) of the low-risk group and the high-risk group. Secondly, the time-dependent receiver operating characteristic (ROC) curve was plotted through the “time ROC” package, and the area under the curve (AUC) values of the OS of the training group samples at 1, 3, and 5 years were calculated to appraise the accuracy of the predictive risk model. Finally, the clinicopathological factors of the two groups and the Risk Score were incorporated into the Cox regression analysis. Packages like “rms” were loaded to construct the nomogram with the variables presenting independent risk factors. The upper part represented the scoring system, and the lower part constituted the prediction system. The 1-year, 3-year, and 5-year survival rates of patients could all be predicted via the scoring system, and the calibration curve and ROC curve were employed to evaluate the accuracy of survival prediction. The GSE42568 + GSE58812 dataset was utilized as the validation set to verify the constructed prediction model.

### 2.6 Assessment of the immunological characteristics of the tumor microenvironment

The infiltration status of 22 distinct immune cells was evaluated by applying packages such as “CIBERSORT” to explore the relationship between the risk score and immune cell infiltration. Additionally, 46 common immune checkpoints were retrieved from relevant literature to investigate the association between the risk score and the 46 immune checkpoint genes. A P value <0.05 indicated a significant threshold.

### 2.7 Drug sensitivity analysis

The expression and drug data of relevant genes were retrieved from the CellMiner database (https://discover.nci.nih.gov/cellminer/home.do). The drug data were filtered through a combined method involving clinical laboratory validation and Food and Drug Administration (FDA) standard certification. Then, the expression data of the prognostic circadian rhythm genes were merged with the drug data, and a Pearson correlation test was conducted to determine the correlation and drug sensitivity between them.

## 3 Result

### 3.1 Differential expression and genetic variation patterns of circadian rhythm genes

**FIGURE 1:**
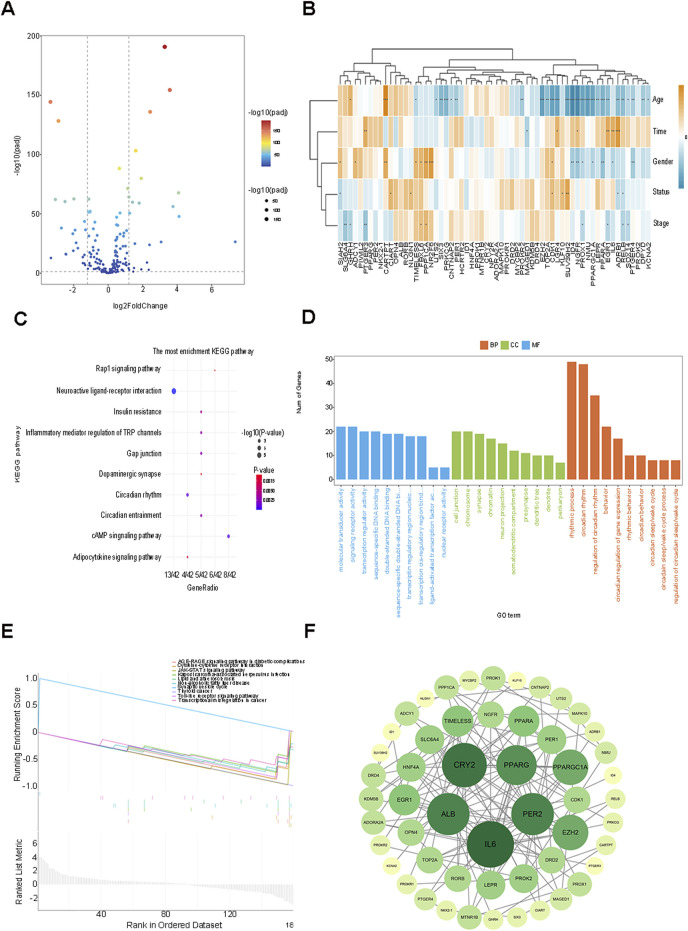
Analysis of circadian rhythm genes in 534 patients with BRCA in the TCGA database. **(A)** 62 DEGs were screened using the TCGA and CGDB databases, encompassing 30 up-regulated genes and 32 down-Regulated genes volcano plot. **(B)** The visualization of the heatmap for the correlation between differential genes and clinical traits. **(C)** GO analysis of the related biological functions and pathways of circadian rhythm genes. **(D)** KEGG analysis of the related biological functions and pathways of circadian rhythm genes. **(E)** Circadian rhythm genes with P < 0.05 were included in the GSEA analysis. **(F)** The Degree algorithm was used to construct a PPI network of 53 nodes to show the interactions among the proteins of circadian rhythm DEGs.

After establishing a one-to-one correspondence between the genetic variation data and the circadian rhythm genes, the data of gene mutations and copy number variations obtained (199 genes, 534 samples) revealed a somatic mutation frequency of 55.43% (296/534 cases) in Breast cancer (BC) (Supplementary Figure S1). The circadian rhythm genes of BC patients collected from the TCGA database were screened for 62 differentially expressed genes (DEGs) with a threshold of P < 0.05 and |log2(Fold Change) | > 1, encompassing 30 upregulated genes and 32 downregulated genes. The visualization results of the volcano plot and the correlation heatmap between the differential genes and clinical traits are presented (Figures 1A, B). Meanwhile, the chromosomal locations of the circadian rhythm DEGs were depicted (Supplementary Figure S2).

### 3.2 Functional enrichment analysis and construction of the differential gene protein interaction network

To explore the pertinent biological functions and pathways of the circadian rhythm genes in BC, GO and KEGG analyses were implemented on the aforementioned 62 differentially expressed genes (DEGs), and the GO classifications and KEGG pathways with the highest enrichment degrees were presented (Figures 1C, D). The GO analysis indicated that the DEGs were primarily enriched in biological functions related to the circadian rhythm process, chromosome, cell junction, and molecular transducer activity, among others. The KEGG analysis mainly manifested enrichment in biological functions associated with neuroactive ligand-receptor interaction pathways, cAMP signaling pathways, Rap1 signaling pathways, and the like. Circadian rhythm genes with P < 0.05 were incorporated into the Gene Set Enrichment Analysis (GSEA) (Figure 1E), and the analysis outcomes were mainly enriched in relevant biological processes such as non-alcoholic fatty liver disease, prion diseases, blood lipids and atherosclerosis, and transcriptional dysregulation in cancer, further validating the intimate relationship between the regulation of genetic material and the metabolism of energy substances such as fatty acids and tumor progression.

Subsequently, after eliminating the DEGs that did not interact with other nodes, a protein-protein interaction (PPI) network of 53 nodes was constructed through the Degree algorithm, revealing the interactions among the circadian rhythm DEGs proteins. The darker the color, the greater the number of nodes associated with the gene (Figure 1F). The results of the above analyses elucidate the relationships at the gene level and also furnish distinct research concepts for identifying potential targets of biomarkers related to prognosis in the future.

### 3.3 Screening of prognostic feature genes based on machine learning algorithms and construction of risk score prediction model

**FIGURE 2:**
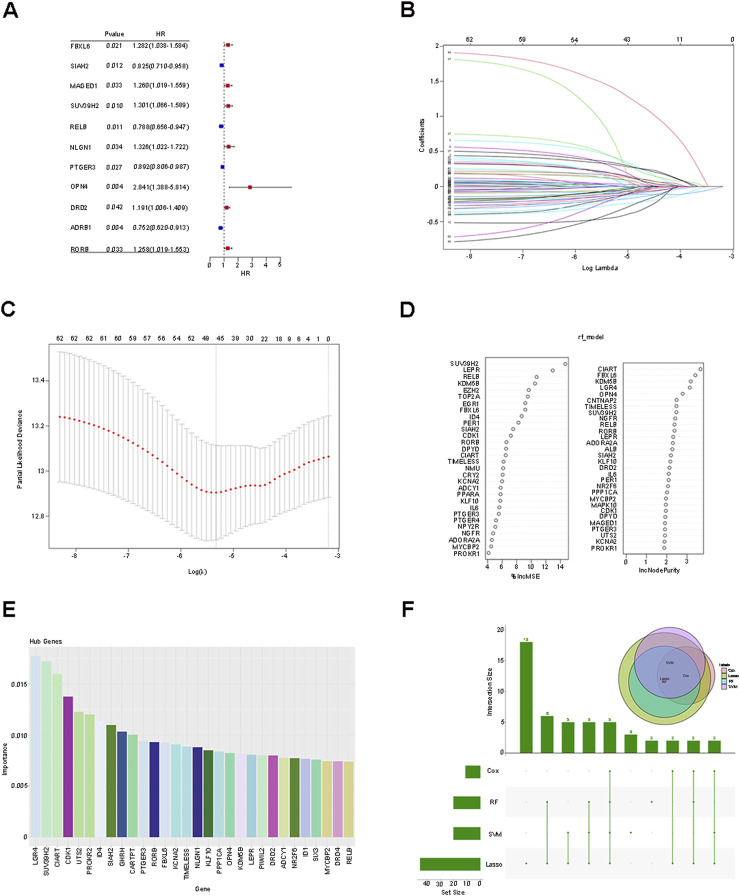
Screening of prognostic characteristic genes and construction of risk score prediction model. **(A)** Cox analysis was employed to analyze 62 differentially expressed genes (DEGs). **(B,C)** Dimension reduction analysis of 62 DEGs was conducted using LASSO regression. **(D)** Random Forest was utilized to analyze 62 DEGs. **(E)** Through the SVM-RFE method, 30 genes with the highest accuracy rate were identified based on their importance scores. **(F)** The intersection among the four methods, namely Cox analysis, LASSO regression, Random Forest, and SVM-RFE, was carried out, and the five prognosis-related characteristic genes after the intersection were visualized as a Venn diagram.

The aforementioned 62 differentially expressed genes (DEGs) were respectively incorporated into Cox analysis, LASSO regression, Random Forest, and SVM-RFE. Cox analysis yielded 11 genes that were significantly associated with independent prognosis (P < 0.05) (Figure 2A). Subsequently, the dimensionality reduction analysis of LASSO regression was carried out, and the minimum value of logλ was indicated by the dotted line, obtaining 46 (seed 10,000) feature genes (Figures 2B, C). Then, after ranking the importance scores of the DEGs by applying Random Forest, 30 candidate genes were identified (Figure 2D), and 30 genes with the highest accuracy were identified by the SVM-RFE method based on their importance scores (Figure 2E). Finally, the DEGs screened by each method (Cox analysis, n = 11; LASSO regression, n = 46; Random Forest, n = 30; SVM-RFE, n = 30) were intersected, and the 5 prognosis-related feature genes (SUV39H2, OPN4, RORB, FBXL6, SIAH2) after taking the intersection were visualized as a Venn diagram (Figure 2F). The RiskScore of each sample was calculated based on the expression levels and risk coefficients of the 5 genes: RiskScore = (Expression level of SUV39H2 × 0.0436) + (Expression level of OPN4 × 1.4270) + (Expression level of RORB × 0.1917) + (Expression level of FBXL6 × 0.3190) + (Expression level of SIAH2 × −0.1984).

### 3.4 The relationship between risk score and clinicopathological characteristics

**FIGURE 3:**
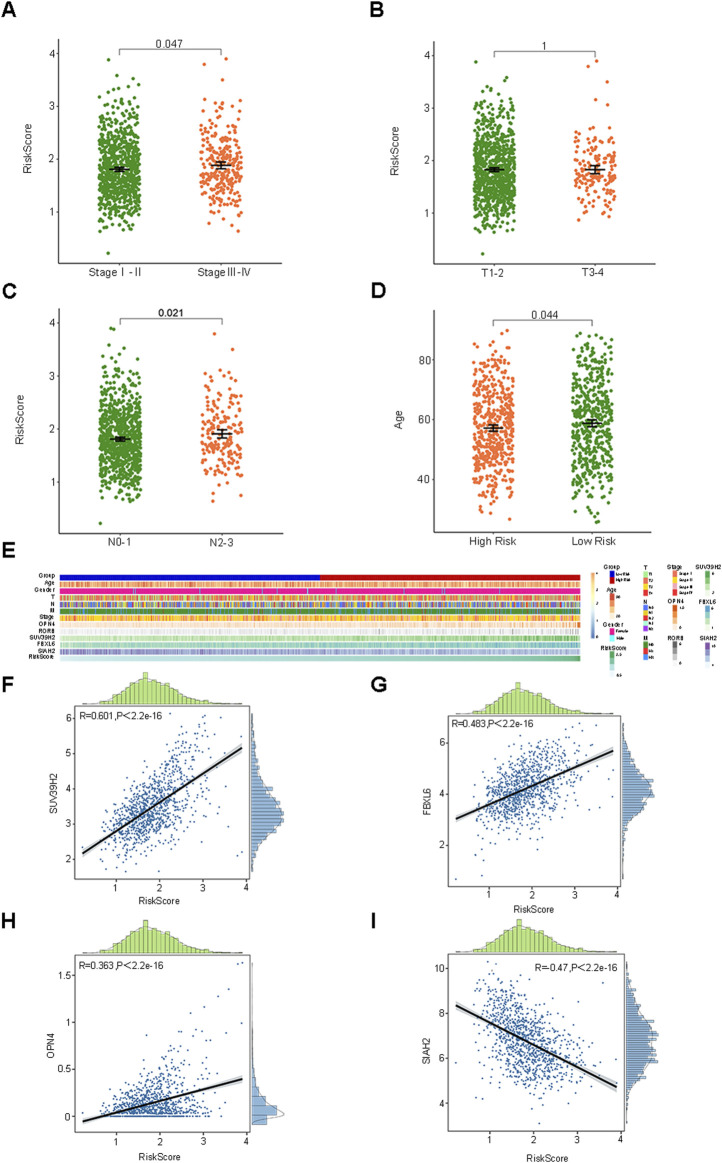
The association of the risk score with clinicopathological features and five prognostic characteristic genes. **(A–E)** Box plots and heat maps depicting the association between risk score and clinicopathological characteristics. **(F–I)** Correlation of risk score with five prognostic characteristic genes.

The median RiskScore of the training group BC samples was employed as the cut-off value to categorize them into the low-risk group (L = 525) and the high-risk group (H = 524). The relationship between the RiskScore and the clinicopathological characteristics was depicted through box plots and heat maps, and patients in different risk groups manifested distinct clinicopathological characteristics (Figures 3A–E). The pathological stage, T stage, and N stage were all correlated with the RiskScore, whereas age and gender were not associated with the RiskScore. Furthermore, we also presented the correlation scatter plots between the RiskScore and the 5 prognostic feature genes (Figures 3F–I, Supplementary Figure S3). SUV39H2, OPN4, RORB, and FBXL6 were positively correlated with the RiskScore, while SIAH2 was negatively correlated with the RiskScore.

### 3.5 Examination of the performance of the risk score prediction model and construction of the nomogram

**FIGURE 4:**
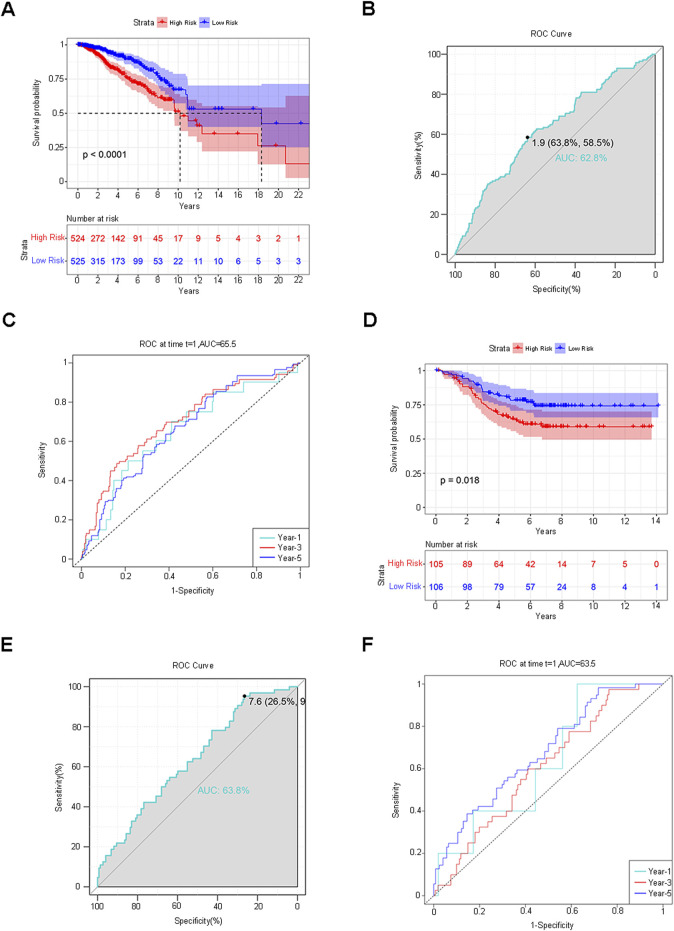
Verification of the Risk Score Prediction Model. **(A)** K-M survival curve: The blue hue represents the low-risk group, while the red hue indicates the high-risk group. **(B,C)** ROC curve and time-dependent ROC curve. The AUC value was computed to offer predictions regarding patient survival. **(D–F)** The identical approach was employed to analyze and validate the dataset. K-M survival curves, ROC curves, and time-dependent ROC curves were delineated.

**FIGURE 5:**
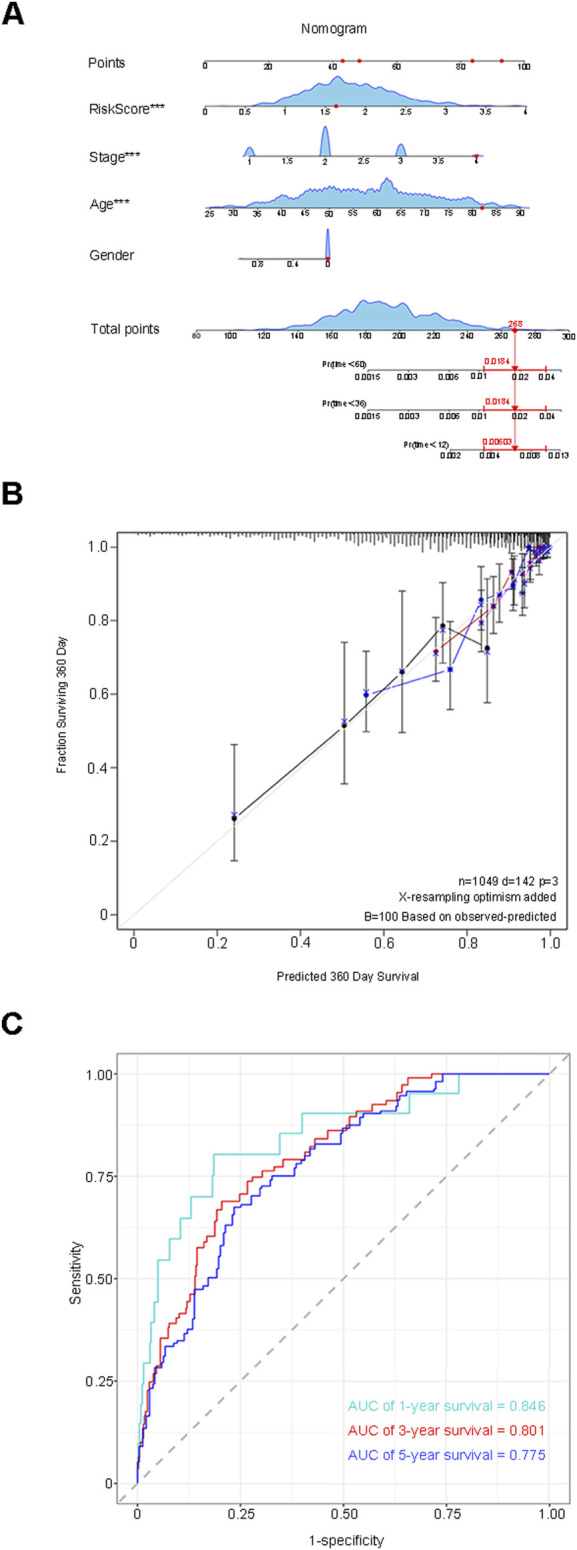
Assess the efficacy of the risk model within clinical practice. **(A)** A Nomogram model was constructed to predict the 1-year, 3-year, and 5-year survival rates of BRCA patients. **(B)** The calibration curve indicates that the results of the Nomogram and the actual observed results exhibit precise overlap in both the training group and the validation group. **(C)** The ROC curve validates the reliability of the prediction of the Nomogram.

The median RiskScore categorized the training group samples into the low-risk group and the high-risk group, and the Kaplan-Meier (K-M) survival curve was plotted. The blue color denoted the low-risk group, and the red color represented the high-risk group. The results demonstrated a statistically significant difference in overall survival (OS) between the two groups (P < 0.0001), suggesting that the high-risk group was more inclined to have an unfavorable prognosis than the low-risk group (Figure 4A). To guarantee the stability of the risk score prediction model, the receiver operating characteristic (ROC) curve and the time-dependent ROC curve were constructed, and the area under the curve (AUC) value was calculated to offer predictions of patient survival (Figures 4B, C). Utilizing the same approach, the samples of the validation group dataset were classified into the low-risk group (L = 106) and the high-risk group (H = 105), followed by the plotting of the K-M survival curve, the ROC curve, and the time-dependent ROC curve. A statistically significant difference in OS persisted between the two groups (p < 0.0001) (Figures 4D–F). The aforementioned results indicated that the model maintained favorable predictive performance in the validation group. By assessing the efficacy of the risk model based on circadian rhythm genes in clinical practice, RiskScore and clinicopathological characteristics were incorporated into the Cox regression analysis to identify variables with independent risk factors, and a Nomogram was developed (Figure 5A). This individualized prediction model could estimate the survival rates of BC patients (1-year, 3-year, and 5-year). It was conspicuously evident from the calibration curve that the outcomes of the Nomogram and the actual observed results exhibited precise overlap in both the training group and the validation group (Figure 5B). The ROC curve also attested that the prediction of the Nomogram was adequately reliable (Figure 5C).

### 3.6 Relationship between risk score and immune cell infiltration

**FIGURE 6:**
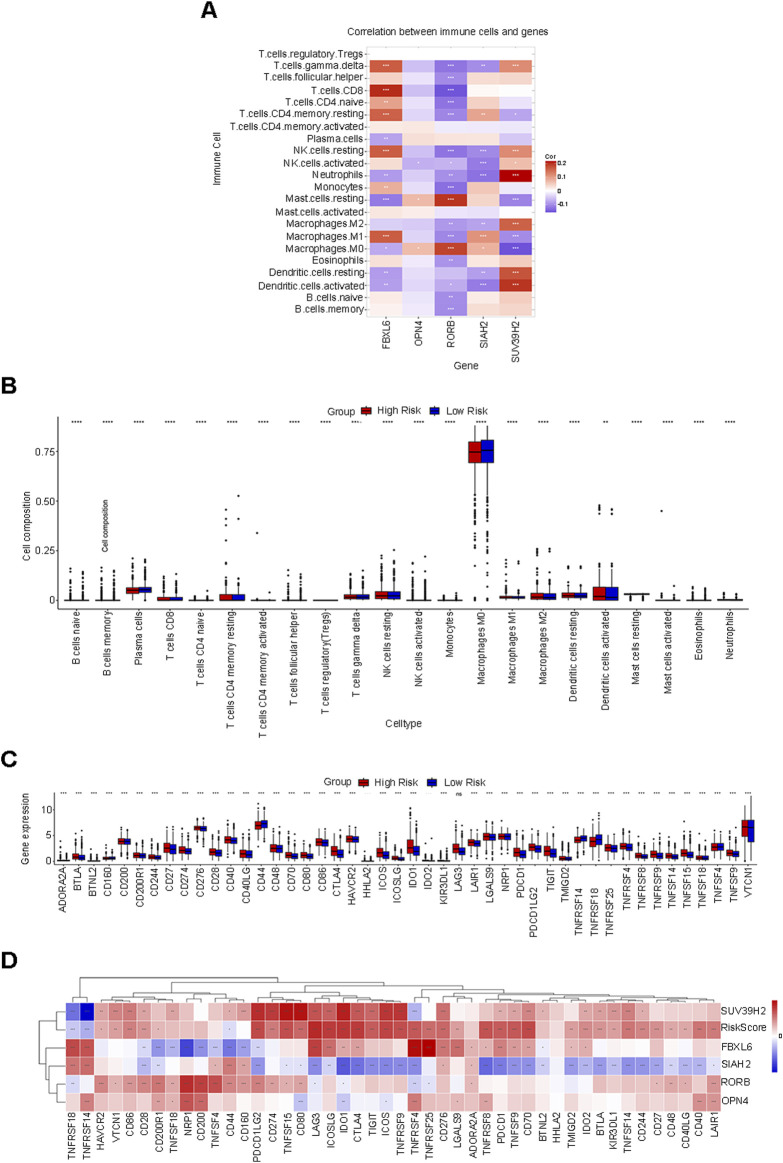
Correlation analysis of the risk score in relation to immune cell infiltration and immune checkpoint genes. **(A)** The correlation between five prognostic characteristic genes and 22 distinct types of immune cells. **(B)** Among the infiltration proportions of 22 types of immune cells, 20 types of immune cells exhibit significant discrepancies between the low-risk group and the high-risk group. **(C)** Expression of immune checkpoint genes in the low-risk and high-risk groups. **(D)** Genes such as SUV39H2, OPN4, RORB, and FBXL6 exhibit a positive correlation with RiskScore, while genes such as SIAH2 demonstrate a negative correlation with RiskScore.

The CIBERSORT algorithm was utilized to compute the correlations between the 5 prognostic feature genes and 22 distinct types of immune cells in order to assess the linkage between the risk score prediction model and immune cell infiltration. In this research, the expression of SUV39H2 was predominantly positively correlated with the infiltration of neutrophils, activated dendritic cells, resting dendritic cells, and M2 macrophages; the expression of SIAH2 was mainly negatively correlated with the infiltration of neutrophils, activated dendritic cells, and activated natural killer (NK) cells; the expression of RORB was mainly positively correlated with the infiltration of resting mast cells and M0 macrophages; the expression of OPN4 was mainly positively correlated with the infiltration of M0 macrophages and resting mast cells; the expression of FBXL6 was mainly positively correlated with the infiltration of CD8 T cells, resting CD4 memory T cells, resting NK cells, M1 macrophages, and γδ T cells (Figure 6A). Furthermore, the levels of immune cell infiltration between the low-risk group and the high-risk group were compared (Figure 6B). Among the infiltration proportions of 22 types of immune cells, 20 immune cells manifested significant discrepancies between the low-risk group and the high-risk group. Particularly, macrophages occupied a considerable proportion in both groups and exhibited significant differences, suggesting that controlling the behavior of this cell is indispensable for intervening in tumor progression in BC patients.

### 3.7 Correlation analysis between risk score and immune checkpoint genes

Within the BC dataset, we delineated the interactions between the risk score and 46 common immune checkpoint genes. The expressions of immune checkpoint genes in the low-risk group and the high-risk group are depicted as follows (Figure 6C). Based on the correlation analysis, genes such as SUV39H2, OPN4, RORB, and FBXL6 were positively correlated with the RiskScore, while genes such as SIAH2 were negatively correlated with the RiskScore (Figure 6D). The aforementioned results might offer potential directions for seeking biological targets related to immunotherapy.

### 3.8 The correlation between the expression of prognostic feature genes and drug susceptibility

**FIGURE 7:**
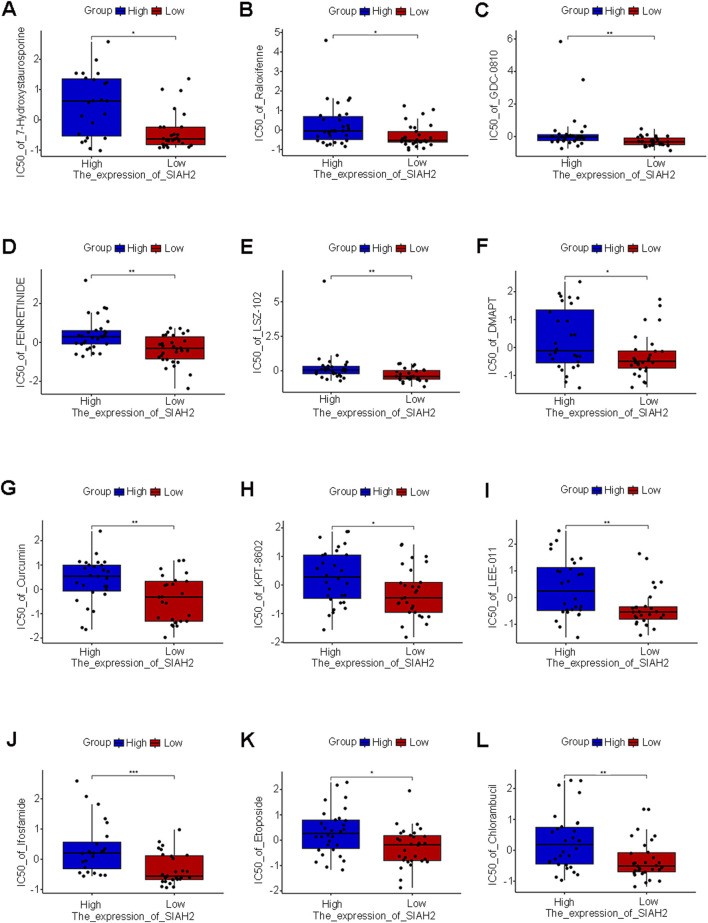
Drug sensitivity of five prognostic characteristic genes. **(A–L)** SIAH2 were performed Pearson correlation analysis with drug data and visualizes the scatter plot of the correlation (p < 0.05).

Pearson correlation analysis was conducted between the five prognostic feature genes and the drug data. The drug sensitivity based on the high and low expression levels of the five prognostic feature genes was calculated (Figures 7A–L, Supplementary Figures S4, 5). We found that the high or low expression of SIAH2 was significantly associated with sensitivity to drugs such as Ifosfamide (p < 0.001). Through analysis, no significant correlations between drug sensitivity and expression were found for the other four genes.

## 4 Discussion

In recent years, the incidence of breast cancer has shown an obvious upward trend, and the age of patients has also shown the characteristics of youthfulness (Gao et al., 2022).

Genetic deficiencies, physical senescence and lifestyle, including shift work systems, jointly or individually contribute to biological rhythm disturbances (Lesicka et al., 2018). In current studies, both animal experiments and population-based epidemiological investigations have demonstrated that biological rhythm disturbances are correlated with an augmented risk of breast cancer occurrence and inferior survival outcomes (Hadadi et al., 2020). To further explore the correlations between circadian rhythm genes and the occurrence, development, prognosis, as well as the tumor microenvironment of breast cancer, we utilized three distinct machine learning algorithms to sift out characteristic circadian rhythm genes and establish a circadian rhythm-related prognostic model.

Firstly, we conducted differential analyses of somatic mutations and copy number variations in BC genetic variation data, obtaining 62 circadian rhythm DEGs, including 30 upregulated genes and 32 downregulated genes, and performed functional enrichment analysis and the construction of PPI. Secondly, Cox analysis, LASSO regression, Random Forest, and SVM-RFE were applied successively to screen the 62 DEGs. Taking the intersection of the DEGs obtained by each method, we ultimately determined the risk score prediction model composed of 5 prognostic characteristic circadian rhythm genes (SUV39H2, OPN4, RORB, FBXL6, SIAH2). Samples were divided into low-risk and high-risk groups based on the median RiskScore. Further research on the prognosis of patients in the two groups showed that the OS of patients in the high-risk group was significantly shorter than that in the low-risk group, and the difference was statistically significant (P < 0.0001). The prognostic prediction performance was evaluated using the ROC curve and the time-dependent ROC curve. The results showed that the AUC value of the ROC curve was 0.628, and the AUC values of the 1-year, 3-year, and 5-year survival rates of the time-dependent ROC curve were 0.655, 0.699, and 0.663, respectively, indicating that the model had good accuracy and was also verified in the external validation dataset (P < 0.0001, 1-year AUC = 0.635, 3-year AUC = 0.607, 5-year AUC = 0.678). Finally, a Nomogram was plotted using RiskScore and the clinicopathological features with independent prognosis in the two groups. This model can accurately predict the 1-year, 3-year, and 5-year survival rates of patients, and its AUC values were 0.846, 0.801, and 0.775 respectively.

The aforementioned five characteristic genes have been reported to be intimately associated with the occurrence and development of various cancers. SUV39H2 (also known as KMT1B), a member of the SUV39 subfamily of lysine methyltransferases (KMT), assumes a critical role in histone H3-K9 dimethylation/trimethylation, transcriptional regulation, and the cell cycle (Li et al., 2019; O'Carroll et al., 2000). Studies have indicated that SUV39H2 is typically overexpressed in cancer tissues, encompassing leukemia, lymphoma, lung cancer, breast cancer, colorectal cancer, gastric cancer, hepatocellular carcinoma, etc., and the dysregulation of SUV39H2 contributes to carcinogenesis and participates in the invasion and metastasis of malignant tumors (Li et al., 2019; Schuhmacher et al., 2015). A growing number of studies have discovered that compared with normal tissues, the expression level of SUV39H2 is significantly elevated in diverse types of cancer tissues and possesses carcinogenic activity. Cancer-related genes such as FAS, P16, P21, Twist1, which mainly function as tumor suppressor genes, are inhibited by the overexpression of SUV39H2, while oncogenes such as PSA and C-myc are enhanced by the overexpression of SUV39H2. SUV39H2 mainly participates in the occurrence and development of cancer as an oncogene, including invasion and metastasis (Li et al., 2019; Shuai et al., 2018; Weirich et al., 2021). FBXL6 is a scarcely studied F-box and leucine-rich repeat sequence (FBXL) protein. Studies have revealed that it can degrade ETV6 (Tel) via the ubiquitin-proteasome system and thereby participates in cell development and differentiation (Li et al., 2021). FBXL6 is overexpressed in various cancers and is correlated with the unfavorable prognosis of the disease, such as gastric cancer, colorectal cancer, hepatocellular carcinoma, and renal cell carcinoma, etc. (Shi et al., 2020; Meng et al., 2023; Yu et al., 2022). OPN4 is a gene closely related to visual perception and circadian rhythm, serving as a light sensor in the circadian rhythm, and it is also an oncogene in cutaneous melanoma (de Assis et al., 2022; Panda et al., 2002). It regulates the cell’s response to UVA radiation by participating in pigmentation, induction of cell death, and molecular clock regulation, resulting in the generation of melanoma (Liu X. et al., 2022). ROR proteins are retinoic acid-related orphan transcription factors belonging to the steroid hormone receptor superfamily and exert regulatory roles in neurogenesis, bone metabolism, and circadian rhythm. Among them, RORB mainly fulfills a role in regulating the biological rhythm (Jetten, 2009; Jetten Am Fau - Ueda and Ueda, 2002). Studies have found that RORB is significantly associated with the risk of various cancers, including breast cancer, prostate cancer, lung cancer, etc. (Mocellin et al., 2018). SIAH2 is a RING E3 ubiquitin ligase, and its overexpression plays a vital role in tumorigenesis and cancer progression (Li et al., 2022). Studies have found that SIAH2 acts as a tumor promoter by targeting key tumor suppressor proteins for degradation, facilitating tumorigenesis in various human malignancies, such as prostate cancer, rectal cancer, breast cancer, and liver cancer, etc. (Li et al., 2022; Liu Q. et al., 2022; Hu et al., 2022; Qi et al., 2010; Qi et al., 2013). Additionally, some other studies have pointed out that SIAH2 also has certain anti-cancer effects (Li et al., 2022).

Based on the aforementioned basis, by implementing the CIBERSORT algorithm to assess the distribution of immune cells between the low-risk group and the high-risk group, it was discovered that macrophages occupied a considerable proportion in both groups. In further analyses, we discovered that the expression of SUV39H2 was mainly positively correlated with the infiltration of neutrophils, activated dendritic cells, resting dendritic cells, and M2 macrophages; the expression of SIAH2 was mainly negatively correlated with the infiltration of neutrophils, activated dendritic cells, and activated natural killer (NK) cells; the expression of RORB was mainly positively correlated with the infiltration of resting mast cells and M0 macrophages; the expression of OPN4 was mainly positively correlated with the infiltration of M0 macrophages and resting mast cells; the expression of FBXL6 was mainly positively correlated with the infiltration of CD8 T cells, resting CD4 memory T cells, resting NK cells, M1 macrophages, and γδT cells. Additionally, the study also found that the expression levels of most immune checkpoint genes were higher in the high-risk group. It can be seen that the expression of characteristic circadian rhythm genes is closely related to the infiltration levels of immune cells. The above analysis results indicate that the activities of immune cells such as macrophages and NK cells are to some extent related to the circadian rhythm, providing a possible direction for subsequent search for potential strategies for treating BC from the perspective of the mechanism by which circadian rhythm disorders affect immune cells.

Through the analysis of drug sensitivity, we discovered that SIAH2 is sensitive to drugs such as Ifosfamide, indicating that SIAH2 can serve as a biological target for drug treatment. The other four genes were not identified as having sensitive drugs in the CellMiner database and still need further in-depth investigation.

Overall, the RiskScore founded on these five genes can be exploited to determine the OS of BC patients. The Nomogram integrating RiskScore and clinical parameters can be utilized to predict the 1-year, 3-year, and 10-year survival rates of BC patients. Hence, this model will facilitate the prognosis and follow-up monitoring of BC patients and offer a reference basis for individualized diagnosis and treatment of BC patients. Meanwhile, we have also verified that the disordered circadian rhythm modifies the infiltration of immune cells in BC patients. Other studies have also manifested that the disordered circadian rhythm is closely related to tumor microenvironment components, immune cell activation, and immune therapeutic responses. This indicates that there is a common phenomenon of circadian rhythm disorder in immune cells within the tumor microenvironment, providing theoretical support for further guiding the research and drug development of the BC mechanism from the perspective of the circadian rhythm immune mechanism in the future. Nevertheless, it should be noted that our study still has certain limitations. Firstly, our research data mainly stem from the TCGA and GEO datasets. The BRCA dataset is unable to support further subtype studies of breast cancer. Another limitation is that the data were sourced from online databases, which did not include information gathered at specific circadian time points. Additionally, it is necessary to assess its predictive efficacy in large independent clinical cohorts. Secondly, we lack in-depth research, especially without designing genome-directed stratified experiments, thus future research experiments are requisite. Subsequently, by expanding the sample size and verifying the applicability of different races, the specific mechanisms of CRRGs and immune therapeutic responses can be further explored using the currently screened five genes (SUV39H2, OPN4, RORB, FBXL6, SIAH2).

## 5 Conclusion

SUV39H2, OPN4, RORB and FBXL6 were positively correlated with Risk Score, while SIAH2 was negatively correlated with Risk Score. The above five circadian rhythm genes can construct a risk model for predicting the prognosis and immune invasion of BC.

## Data Availability

The datasets presented in this study can be found in online repositories. The names of the repository/repositories and accession number(s) can be found in the article/Supplementary Material.

## Supplementary material

SUPPLEMENTARY FIGURE S1 The somatic mutation frequency of circadian rhythm genes in 534 BRCA patients within the TCGA database.

SUPPLEMENTARY FIGURE S2 The locations of circadian rhythm DEGs on chromosomes.

SUPPLEMENTARY FIGURE S3 Correlation of risk score with RORB gene.

SUPPLEMENTARY FIGURE S4 SUV39H2, OPN4, FBXL6 and SIAH2 were performed Pearson correlation analysis with drug data and visualizes the scatter plot of the correlation (p>0.05).

SUPPLEMENTARY FIGURE S5 RORB was performed Pearson correlation analysis with drug data and visualizes the scatter plot of the correlation (p>0.05).
