# Supplementary figures and images for: Construction of a circadian rhythm-related gene signature for predicting the prognosis and immune infiltration of breast cancer
Source: Front Mol Biosci. 2025 Feb 6;12:1540672. doi: 10.3389/fmolb.2025.1540672 (PMC11839441; doi:10.3389/fmolb.2025.1540672)

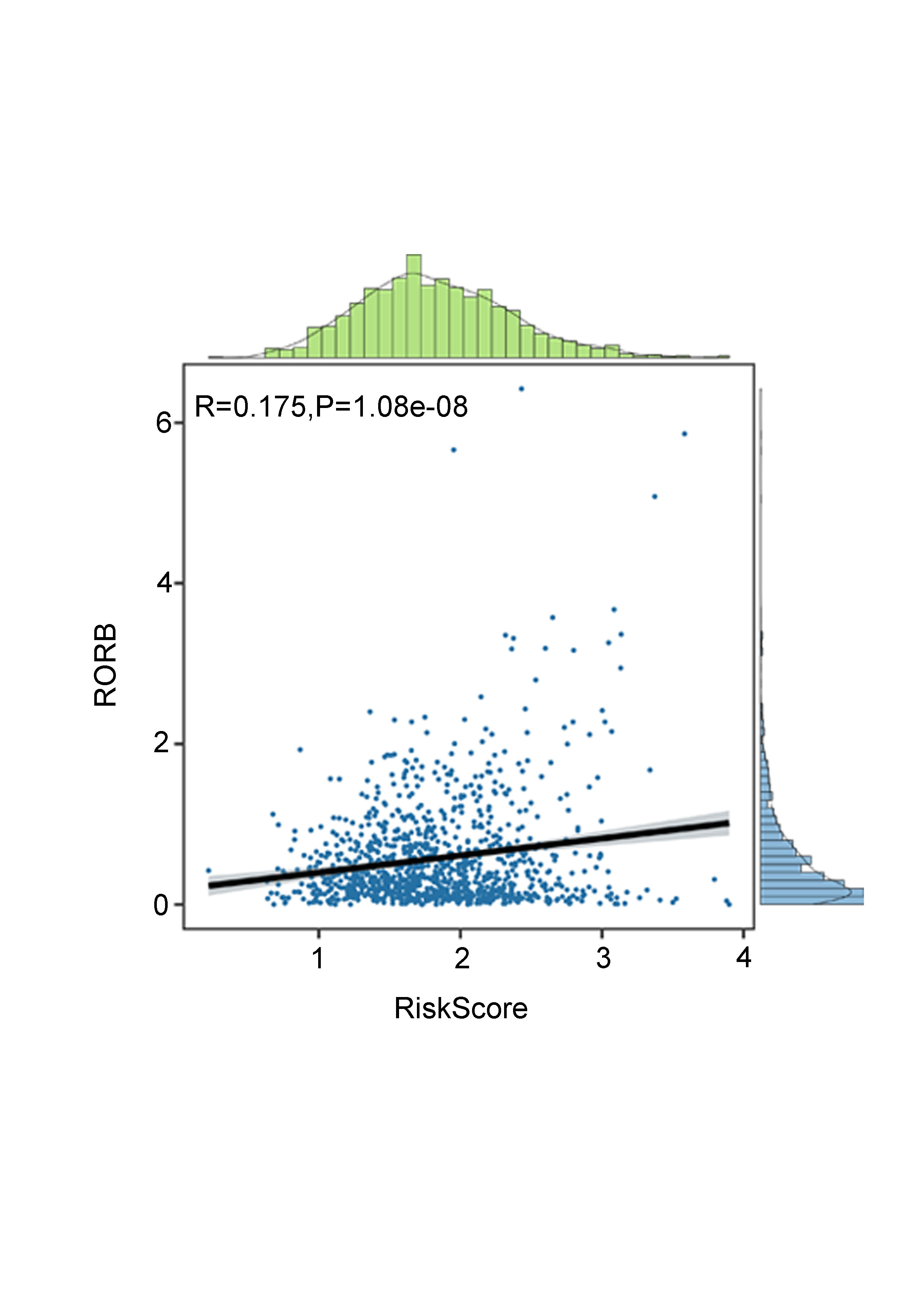

Supplement: Supplementary file 1 [file Image3.tif]

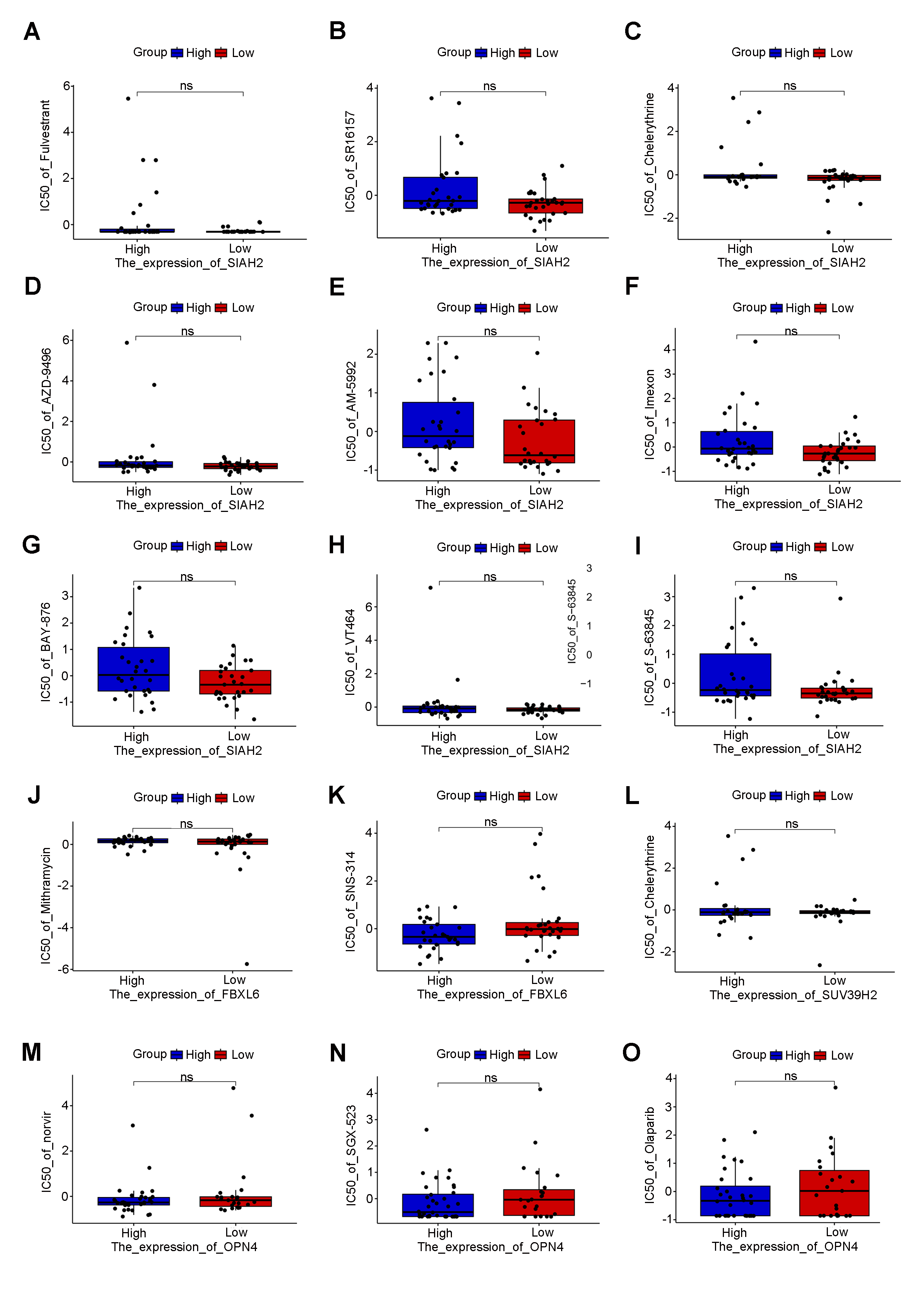

Supplement: Supplementary file 2 [file Image4.tif]

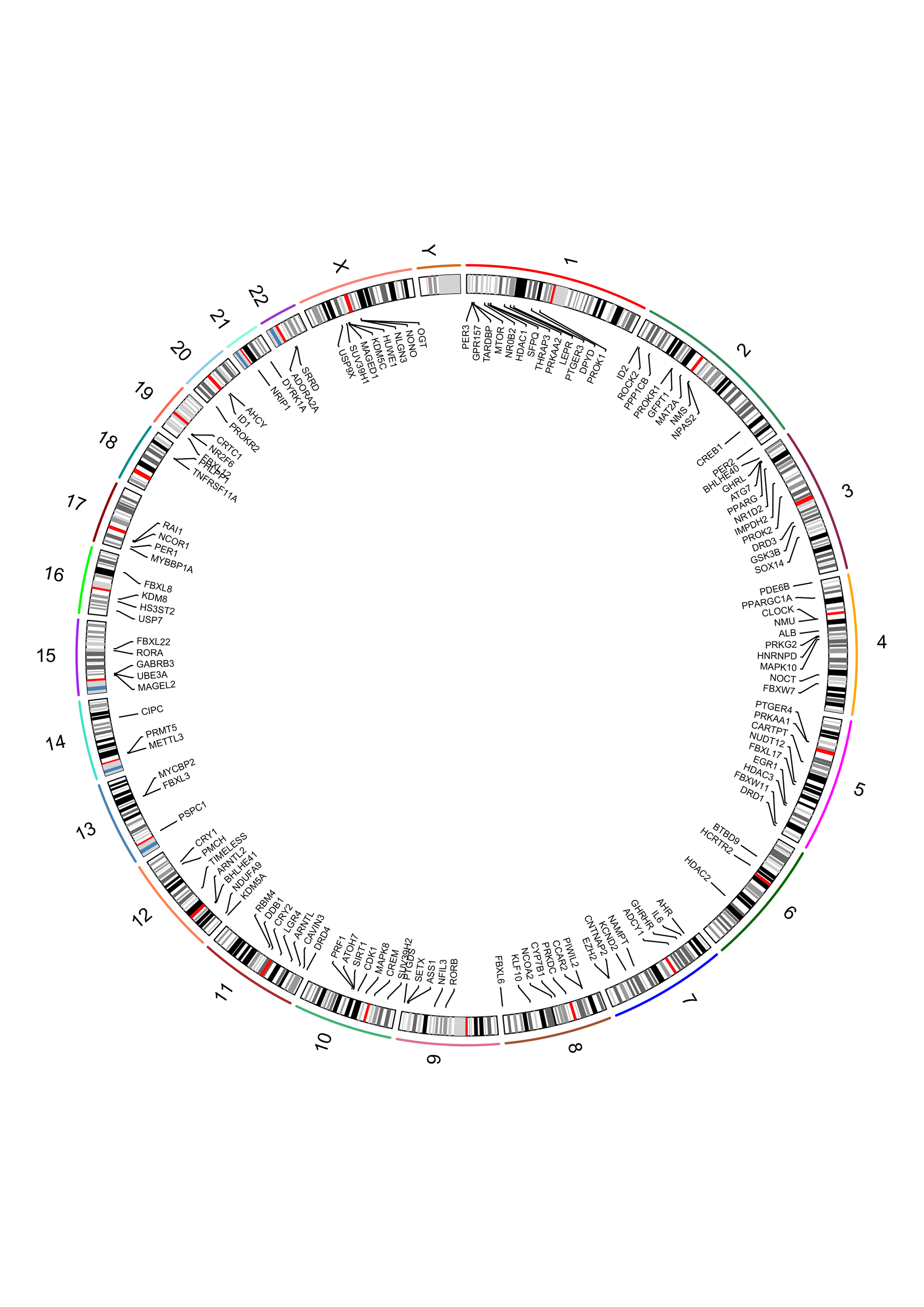

Supplement: Supplementary file 3 [file Image2.tif]

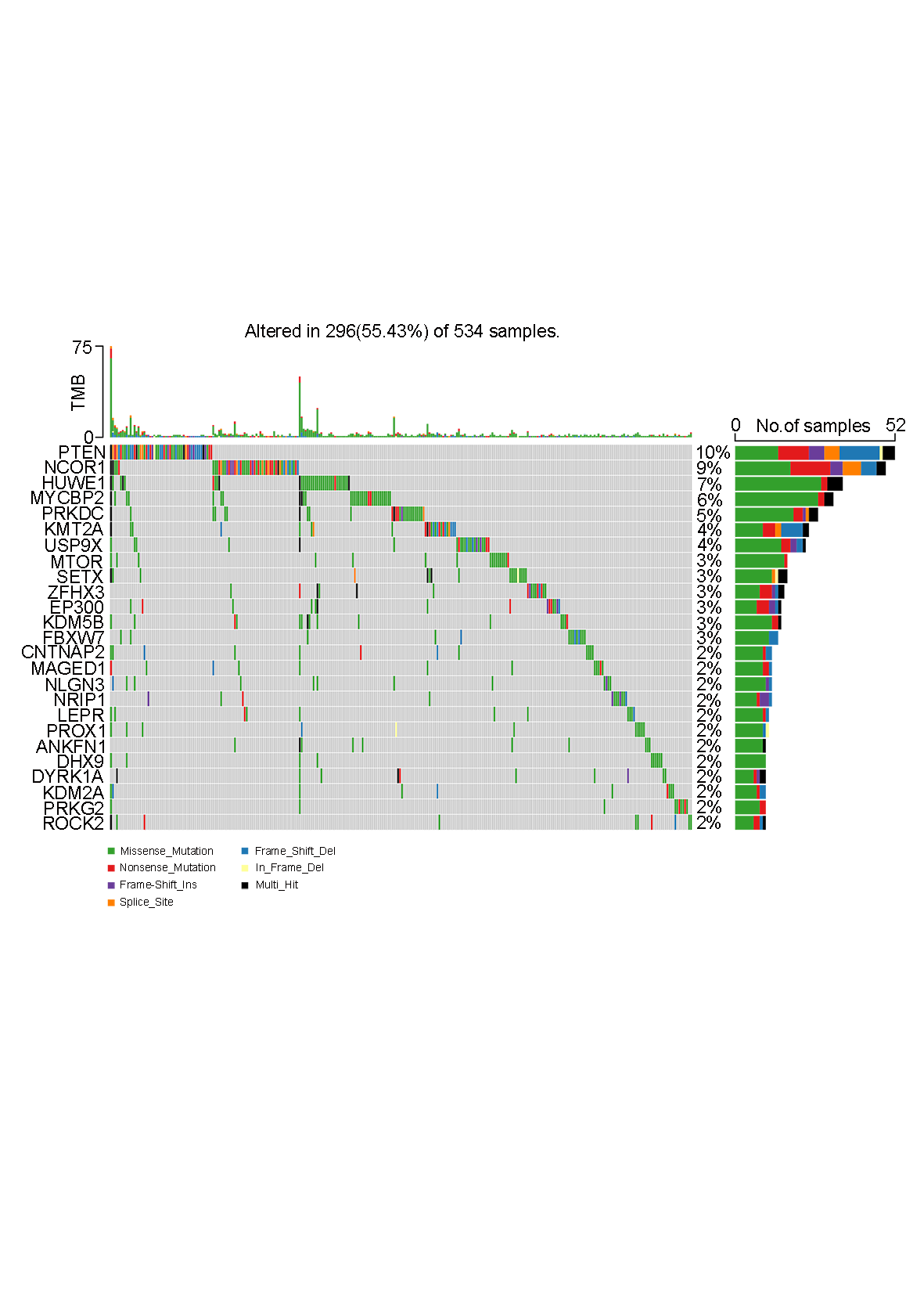

Supplement: Supplementary file 4 [file Image1.tif]

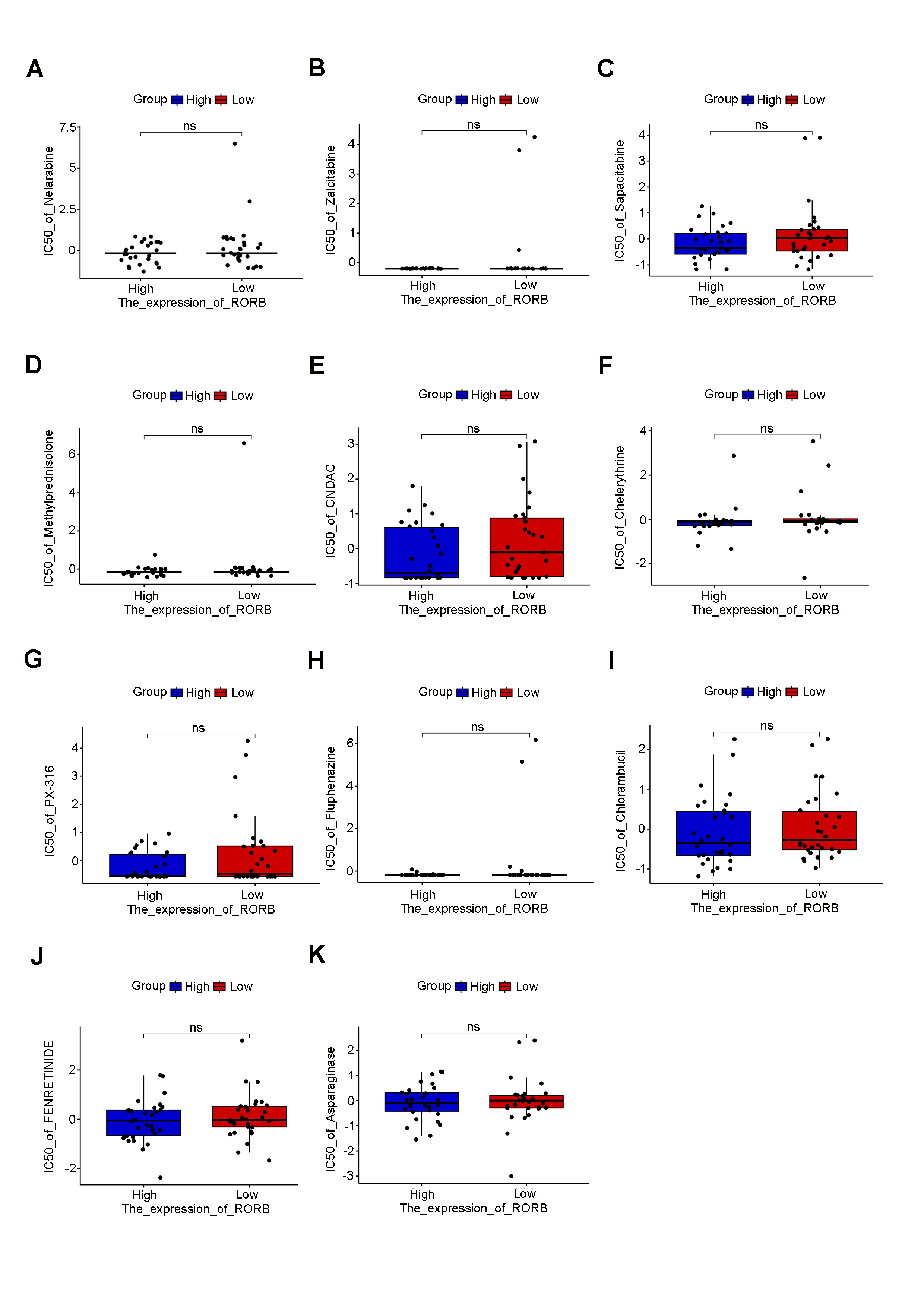

Supplement: Supplementary file 5 [file Image5.tif]
